# Activation of activator protein 2 alpha by aspirin alleviates atherosclerotic plaque growth and instability *in vivo*

**DOI:** 10.18632/oncotarget.10400

**Published:** 2016-07-04

**Authors:** Jing-Jing Yang, Peng Li, Fu Wang, Wen-Jing Liang, Hui Ma, Yuan Chen, Zhi-Min Ma, Quan-Zhong Li, Qi-Sheng Peng, Yun Zhang, Shuang-Xi Wang

**Affiliations:** ^1^ Key Laboratory of Cardiovascular Remodeling and Function Research, Qilu Hospital, School of Medicine, Shandong University, Jinan, China; ^2^ Department of Pharmacology, College of Pharmacy, Xinxiang Medical University, Xinxiang, China; ^3^ Division of Endocrinology, The Second Affiliated Hospital, Soochow University, Suzhou, China; ^4^ Division of Cardiology, The Affiliated Hospital, Guilin Medical University, Guilin, China; ^5^ Key Laboratory for Zoonosis Research, Ministry of Education, Institute of Zoonosis, Jilin University, Changchun, China

**Keywords:** activator protein 2α, aspirin, IkBα, atherosclerosis, Pathology Section

## Abstract

**Aims:**

Aspirin has been used for the secondary prevention and treatment of cardiovascular disease for several decades. We investigated the roles of transcriptional factor activator protein 2α (AP-2α) in the beneficial effects of aspirin in the growth and vulnerability of atherosclerotic plaque.

**Methods and Results:**

In mice deficient of apolipoprotein E (*Apoe^-/-^*), aspirin (20, 50 mg/kg/day) suppressed the progression of atherosclerosis in aortic roots and increased the plaque stability in carotid atherosclerotic plaques induced by collar-placement. *In vivo* lentivirus-mediated RNA interference of AP-2α reversed the inhibitory effects of aspirin on atherosclerosis in *Apoe^-/-^* mice. Mechanically, aspirin increased AP-2α phosphorylation and its activity, upregulated IkBα mRNA and protein levels, and reduced oxidative stress in cultured vascular smooth muscle cells. Furthermore, deficiency of AP-2α completely abolished aspirin-induced upregulation of IkBα levels and inhibition of oxidative stress in *Apoe^-/-^* mice. Clinically, conventional doses of aspirin increased AP-2α phosphorylation and IkBα protein expression in humans subjects.

**Conclusion:**

Aspirin activates AP-2α to upregulate IkBα gene expression, resulting in attenuations of plaque development and instability in atherosclerosis.

## INTRODUCTION

Atherosclerosis, formerly considered a bland lipid storage disease, actually involves an ongoing inflammatory response [1]. In the early stage of atherosclerosis, the abnormal homeostatic functions of the endothelium appear and promote an inflammatory response. Later in the process, increased inflammation stimulates enlargement of the plaque lesion weakening the protective fibrous cap of the atheroma [2], possibly leading to thrombosis and the occurrence of acute coronary syndrome.

Activating enhancer binding protein 2 alpha, also called activator protein 2α (AP-2α), is a member of the AP-2 transcription factor family proteins consisting of α, β, γ, δ, and ε subunits [3]. Mice deficient in AP-2 die after birth due to the abnormal skeletal and neural tube development, indicating a critical role of AP-2 in mammals [4, 5]. AP-2α recognizes the consensus DNA sequence of 5′-GCCNNNGGC-3′ found in a number of genes involved in various cell functions [6]. The biological role and clinical relevance of AP-2α in atherosclerosis have not been elucidated.

The ancient drug aspirin reduces the risks of cardiovascular disease (CVD) in part by its anti-inflammatory and antiplatelet effects via the inhibition of cyclooxygenase (COX) enzymes [7]. The efficacy of aspirin for secondary prevention of CVD, such as atherosclerosis, is well established, but the benefit of aspirin for primary prevention is controversial [8-10].

Aspirin activates AMP-activated protein kinase (AMPK) in vascular cells [11-14]. Previously, we have shown that activation of AMPK increases AP-2α phosphorylation in vascular smooth muscle cells (VSMC) and suppresses the formation of atherosclerotic plaques in mice [15-17]. Based on these studies, we hypothesize that aspirin induces AP-2α phosphorylation leading to attenuation of the progression of atherosclerosis, including the growth and instability of plaque. In this study, we provided evidence that AP-2α is crucial to the anti-inflammatory effects of aspirin that are independent of COX. Given the significant effects of aspirin-induced AP-2α activation on plaque growth and stability, AP-2a may be an attractive target for the prevention of diseases related to the rupture of atherosclerotic plaques, such as stroke or myocardial infarction clinically.

## RESULTS

### Aspirin attenuates the growth of atherosclerotic plaque in *Apoe* mice

**Figure 1:**
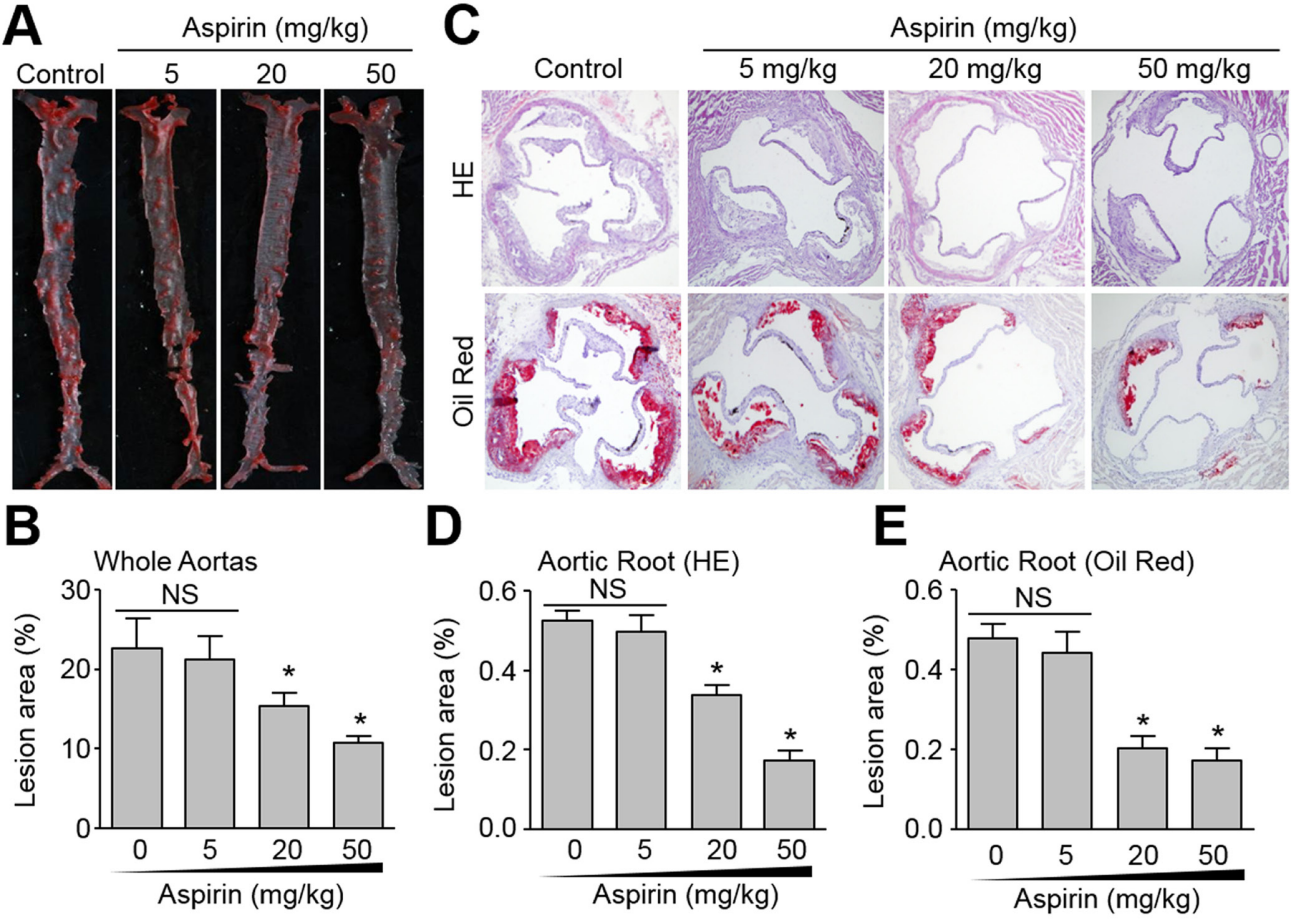
Effects of aspirin on aortic atherosclerotic plaque growth in *Apoe^-/-^* mice The protocol and experimental designs were described in Supplementary Methods and Figure S1A. After 8-weeks of aspirin treatment, all mice were sacrificed under anaesthesia. **A.** The whole aortas including thoracic and abdominal aortas were collected for histological analysis of atherosclerosis by Oil Red staining of lipid deposition. **B.** Quantitative data of atherosclerotic lesion in the whole aortas. C. Histological analysis of aortic root by HE staining and Oil Red (original magnification X100). **D.** Quantitative analysis of atherosclerotic lesion size in aortic root by HE staining. **E.** Quantitative analysis of atherosclerotic lesion size in aortic root by Oil Red staining. N is 10-15 in each group. *P < 0.05 *vs*. Control (Aspirin at 0). NS indicates no significant difference.

Aspirin is used to reduce the risk of CVD in secondary prevention [18]. We firstly tested the dose-response of aspirin (5, 20, or 50 mg/kg/day) on the suppression of atherosclerotic lesion formation in *Apoe^-/-^* mice fed a high fat diet for 8 weeks as described previously [19]. The atherosclerotic plaques in whole aortas (Figure 1A, 1B) and aortic roots (Figure 1C-1E) were determined by Oil Red staining or HE staining. As indicated in Figure 1A-1E, 5 mg/kg/day aspirin did not reduce the size of atherosclerotic plaque, consistent with previous reports indicating that this dose of aspirin is ineffective [9]. However, 20 and 50 mg/kg/day aspirin significantly decreased the size of atherosclerotic plaques in whole aortas and aortic roots, consistent with a recent report [20]. An aspirin dose response was evident with 50 mg/kg/day aspirin substantially suppressing atherosclerotic formation compared to 20 mg/kg/day aspirin. These data indicate that aspirin has the potential to produce anti-atherosclerotic effect *in vivo*.

### Aspirin increases the stability of atherosclerotic plaque in *Apoe^-/-^* mice

**Figure 2:**
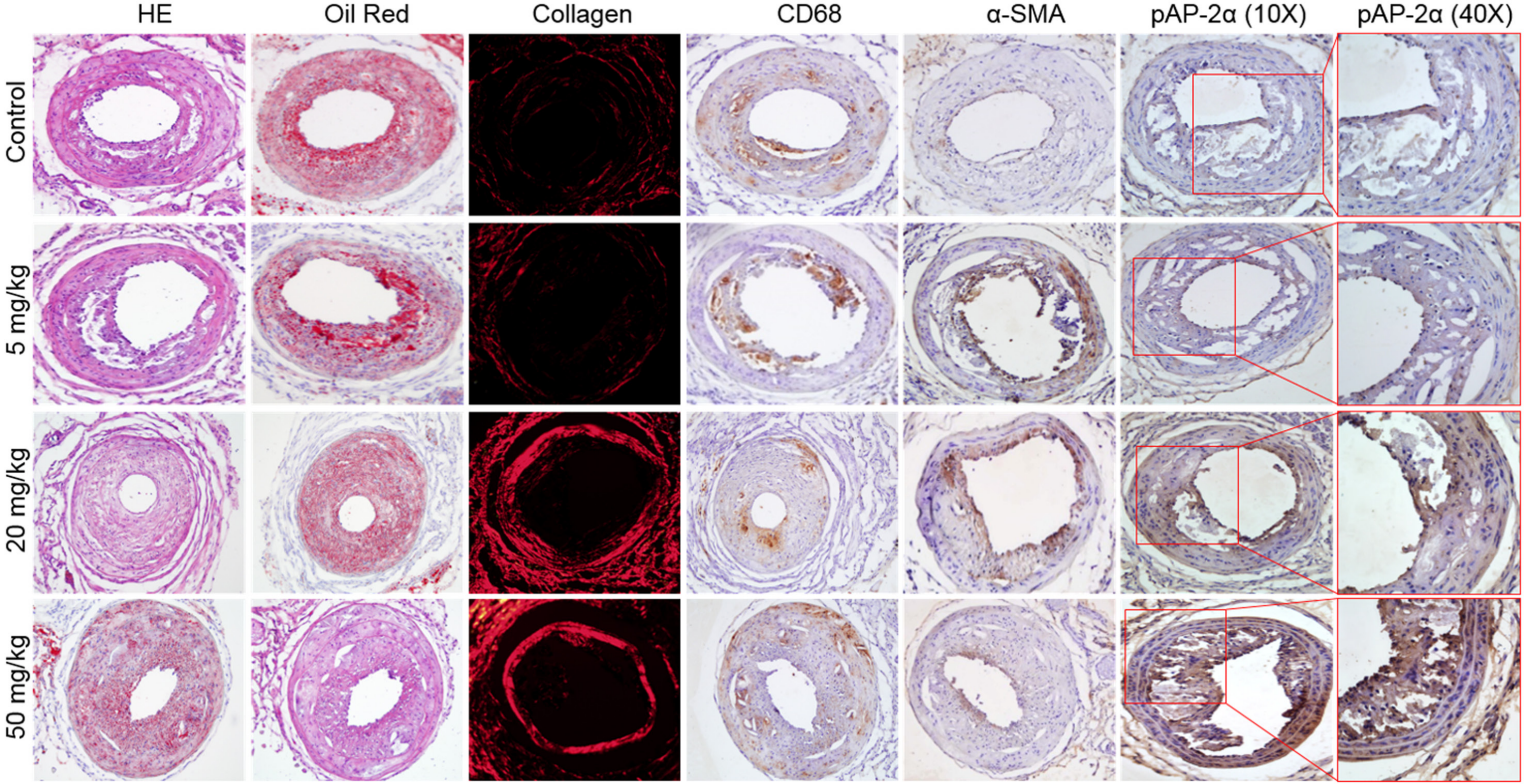
Effects of aspirin on carotid atherosclerotic plaque stability in *Apoe^-/-^* mice The protocol and experimental designs were described in Supplementary Methods and Figure S1A. After 8-weeks of aspirin treatment, all mice were sacrificed under anaesthesia. Histological analysis of left common carotid arteries by HE staining, lipid content by Oil Red staining, collagen content by picrosirius red, and IHC analysis of CD68, α-SMA, and phosphorylated AP-2α (p AP-2α) (original magnification X100). 10-15 mice in each group. All quantitative data were shown in Supplementary Figure S1B-S1H.

In advanced atherosclerosis, rapture of instable atherosclerotic plaques contributes to a large number of victims of coronary heart disease [21]. Thus, we next examined the effects of aspirin on the stability of high-risk prone-to-rupture plaques. The carotid collar model of vulnerable plaque induction was performed in *Apoe^-/-^* mice by placing a collar around the left common carotid artery and feeding the mice a high fat diet for 12 weeks as describe previously [22]. Aspirin was introduced into the drinking water from 4 to 12 weeks. HE staining of the left carotid artery revealed similar plaque lesion areas independent of aspirin treatment (Figure 2 and Supplementary Figure S1B), allowing atherosclerotic plaque stability to be evaluated. As shown in Figure 2 and Supplementary Figure S1C-S1F, aspirin at 20 or 50 mg/kg/day, but not 5 mg/kg/day, significantly decreased the levels of infiltrating CD68 positive macrophages, Oil Red O staining, and increased expression of collagen and α-smooth muscle actin (α-SMA) as indicators of VSMC and stability of carotid atherosclerotic plaques. Statistically, both doses of aspirin (20, 50 mg/kg/day) decreased the plaque vulnerable index (Supplementary Figure S1G), which was calculated according to the ratio of area I (Oil Red^+^ + CD68^+^) to area II (α-SMA^+^ + Collagen^+^) as described previously [23, 24]. 50 mg/kg/day aspirin was more effective than 20 mg/kg/day at maintaining the stability of vulnerable plaques. Our results suggests that aspirin increases plaque stability in advanced vulnerable atherosclerosis.

### Aspirin increases phosphorylation of AP-2α in *Apoe^-/-^* mice

Aspirin has been reported to activate AMPK [11]. Our previous studies revealed that AMPK directly phosphorylates AP-2α at serine 219, an active form if it is phosphorylated [17]. Thus, we hypothesized that aspirin is able to activate AP-2α *via* serine 219 phosphorylation. To test this notion, we determined the effects of aspirin on AP-2α serine 219 phosphorylation in the carotid model of plaque development. As presented in Figure 2 and Supplementary Figure S1H, 20-50 mg/kg/day aspirin, but not 5 mg/kg/day, remarkably increased AP-2α phosphorylation *in vivo*, supporting an important role of AP-2α in the protective effects of aspirin on atherosclerosis. Since the effects of 50 mg/kg/day aspirin on atherosclerotic plaque stability and AP-2α phosphorylation were maximal, we chose 50 mg/kg/day as the optimal dose in most of the following animal experiments.

### Lentivirus-mediated RNA interfere of AP-2α abolishes the suppressive effects of aspirin on atherosclerotic plaque growth in *Apoe^-/-^* mice

**Figure 3:**
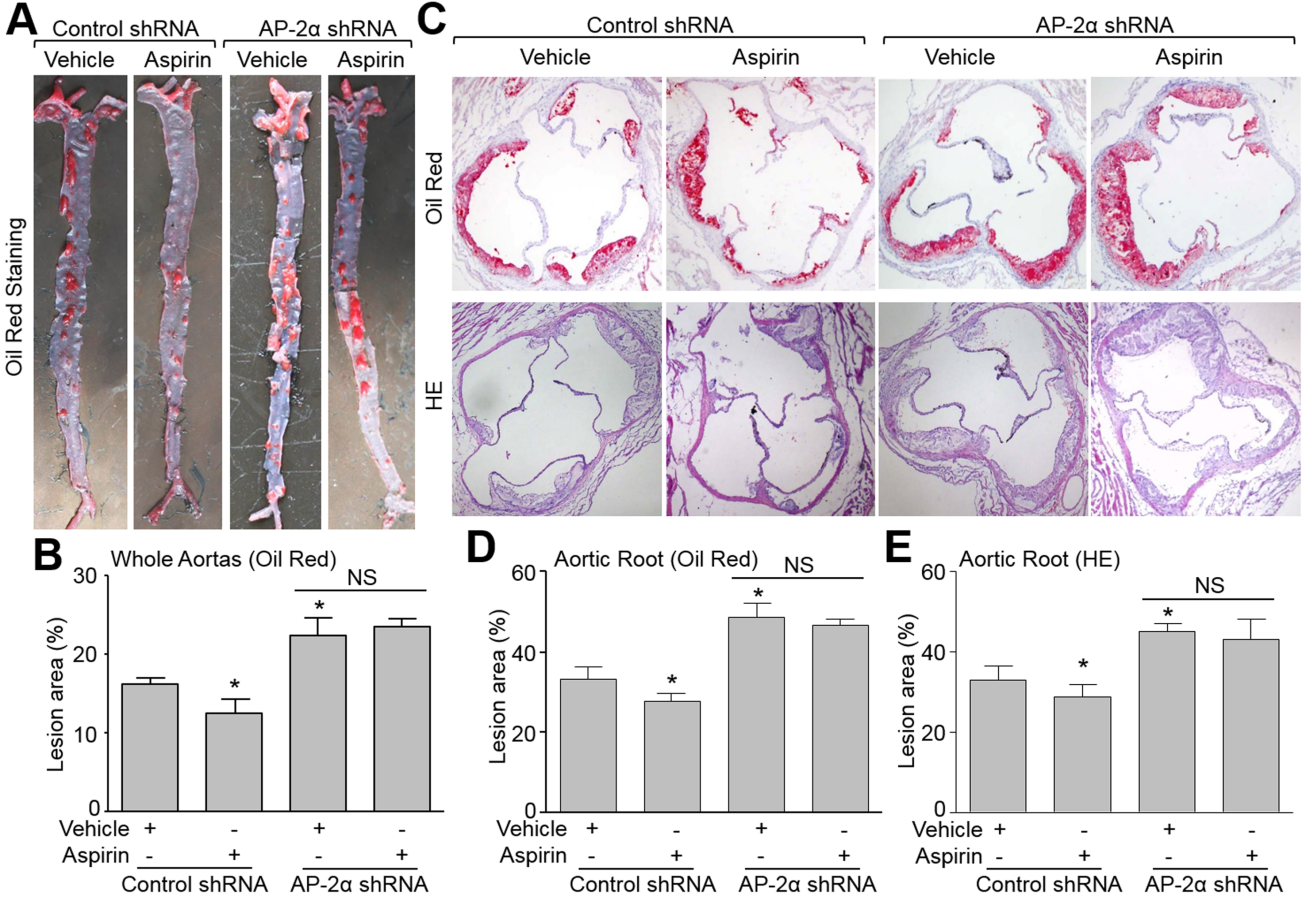
Deficiency of AP-2α abolishes the suppressive effects of aspirin on aortic atherosclerotic lesion development in *Apoe^-/-^* mice The protocol and experimental designs were described in Supplementary Methods and Figure S2A. **A.** Morphological analysis of atherosclerotic lesions in whole aortas by Oil Red staining. **B.** Quantitative data of atherosclerotic lesions. 10-15 mice in each group. *P < 0.05 *vs*. control shRNA alone. NS indicates no significance. **C.** Morphological analysis of aortic roots by Oil Red or HE staining (original magnification X100). **D.** Quantitative analysis of atherosclerotic lesion size in aortic roots by Oil Red in C. **E.** Quantitative analysis of atherosclerotic lesion size in aortic roots by HE in C. Positive stained area is given as the percentage of the totally analyzed area of the aorta. N is 10-15 in each group. *P < 0.05 *vs.* control shRNA alone. NS indicates no significance.

We next examined the roles of AP-2α in the suppressive effects of aspirin on atherosclerosis in *Apoe^-/-^*mice. Due to the *lethal* phenotype of mice deficient in AP-2α [5], we generated AP-2α-knockdown mice by infecting lentivirus containing AP-2α short hairpin RNA (shRNA) to downregulate AP-2α protein expression in *Apoe^-/-^* mice (Supplementary Figure S2B). AP-2α shRNA lentivirus did not alter metabolic characters including TG, TC, BG, and blood monocyte counts (Supplementary Table S1). As depicted in Figure 3A, 3B, and 3E, AP-2α shRNA treated mice had significantly higher lesion areas in the aorta and aortic arch than control shRNA treated mice. Aspirin significantly suppressed whole aortas and aortic arch lesion areas in *Apoe^-/-^* mice infected with control shRNA lentivirus, but had no significant suppression in *Apoe^-/-^*- mice infected with AP-2α shRNA lentivirus. Similarly, aspirin decreased the Oil Red staining of lipids in the aortic arch of control shRNA treated in *Apoe^-/-^* mice but did not in AP-2α shRNA lentivirus treated mice (Figure 3C, 3D). These data demonstrate that AP-2α is required for atherosclerotic plaque suppression by aspirin *in vivo*.

### Knockdown of AP-2α ablates the effects of aspirin on maintaining atherosclerotic plaque stability in *Apoe^-/-^* mice

**Figure 4:**
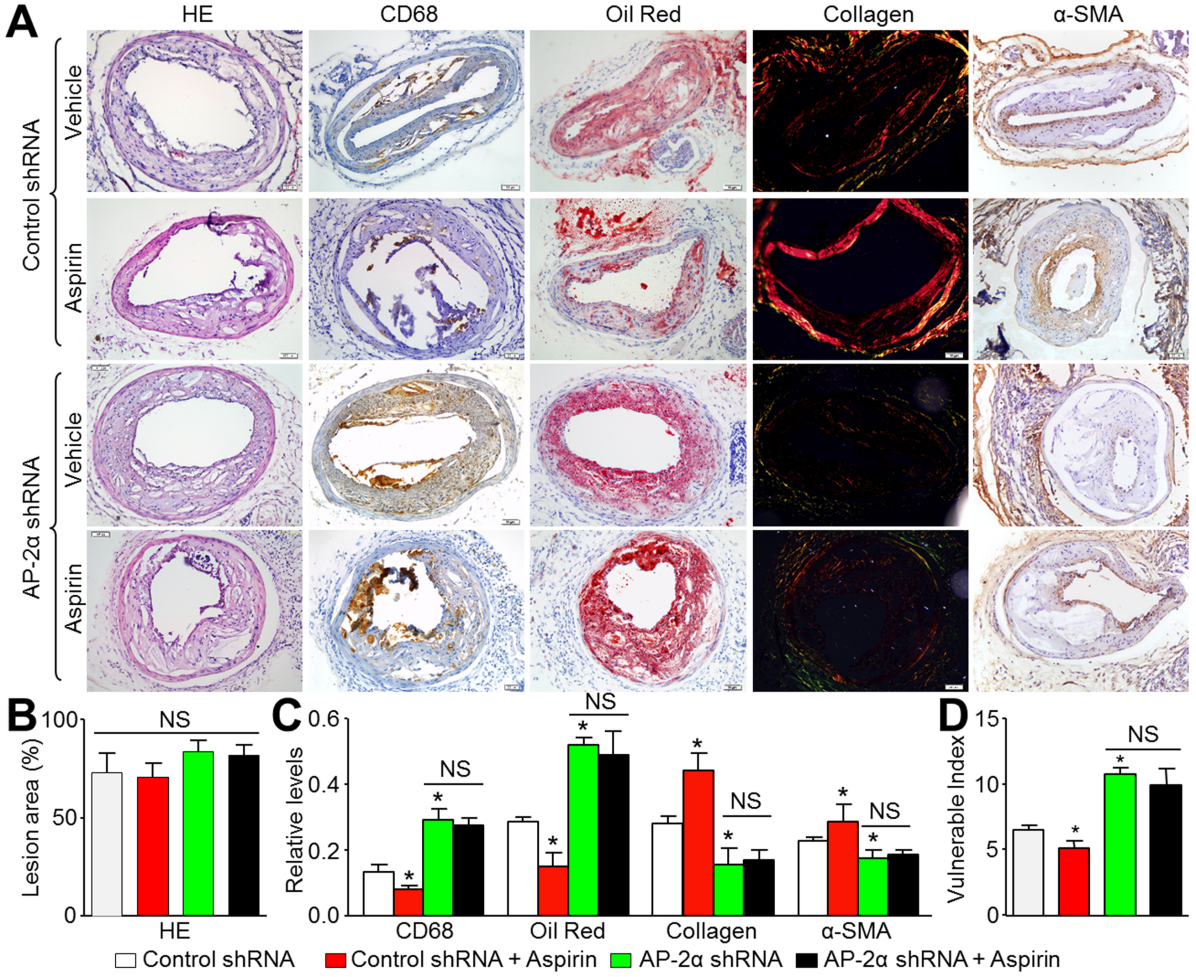
Aspirin increases carotid atherosclerotic plaque stability in *Apoe^-/-^* mice *via* AP-2α The protocol and experimental designs were described in Supplementary Methods and Figure S2C. **A.** Morphology of left common carotid arteries by HE staining, Oil Red staining for lipid content, picrosirius red staining of collagen, numbers of vascular smooth muscle cells by α-SMA, and macrophages by CD68, respectively (original magnification X100). **B.** Quantitative data for sizes of atherosclerotic lesion in left common carotid arteries. **C.** Quantitative data for lipid content, collagen, VSMC number, and macrophages number. **D.** Vulnerability index was determined by using the ration of CD68^+^ (%) plus Oil Red (%) to α-SMA (%) plus collagen (%). N is 10-15 in each group. *P < 0.05 *vs*. control shRNA alone. NS indicates no significant difference.

We next determined the role of AP-2α in aspirin-induced atherosclerotic plaque stability using the carotid collar model of vulnerable plaque formation in *Apoe^-/-^* mice treated with control or AP-2α shRNA lentivirus. AP-2α shRNA lentivirus -mediated RNA interference significantly inhibited AP-2α protein expression in the plaque area of carotid arteries compared to control lentivirus (Supplementary Figure S2D and S2E). Vulnerable plaque sizes induced by collar placement were comparable in all treatment groups *Apoe^-/-^* mice (Figure 4A, 4B). Histological analysis revealed that aspirin increased the levels of α-SMA and collagen in the plaque, and reduced the levels of lipid and CD68 in *Apoe^-/-^* mice treated with control shRNA (Figure 4A, 4C). By calculating plaque vulnerable index, aspirin increased the plaque stability in *Apoe^-/-^* mice (Figure 4D). However, AP-2α shRNA knockdown of AP-2α induced significantly increased CD68 and lipid deposition as well as suppressed collagen and α-SMA expression indicating highly vulnerable plaque formation (Figure 4A, 4C, 4D). Aspirin could not suppress plaque instability in the AP-2α shRNA treated mice. These data demonstrate that AP-2α is essential for atherosclerotic plaque stability induced by aspirin.

### Aspirin increases AP-2α DNA-binding activity in VSMCs

**Figure 5:**
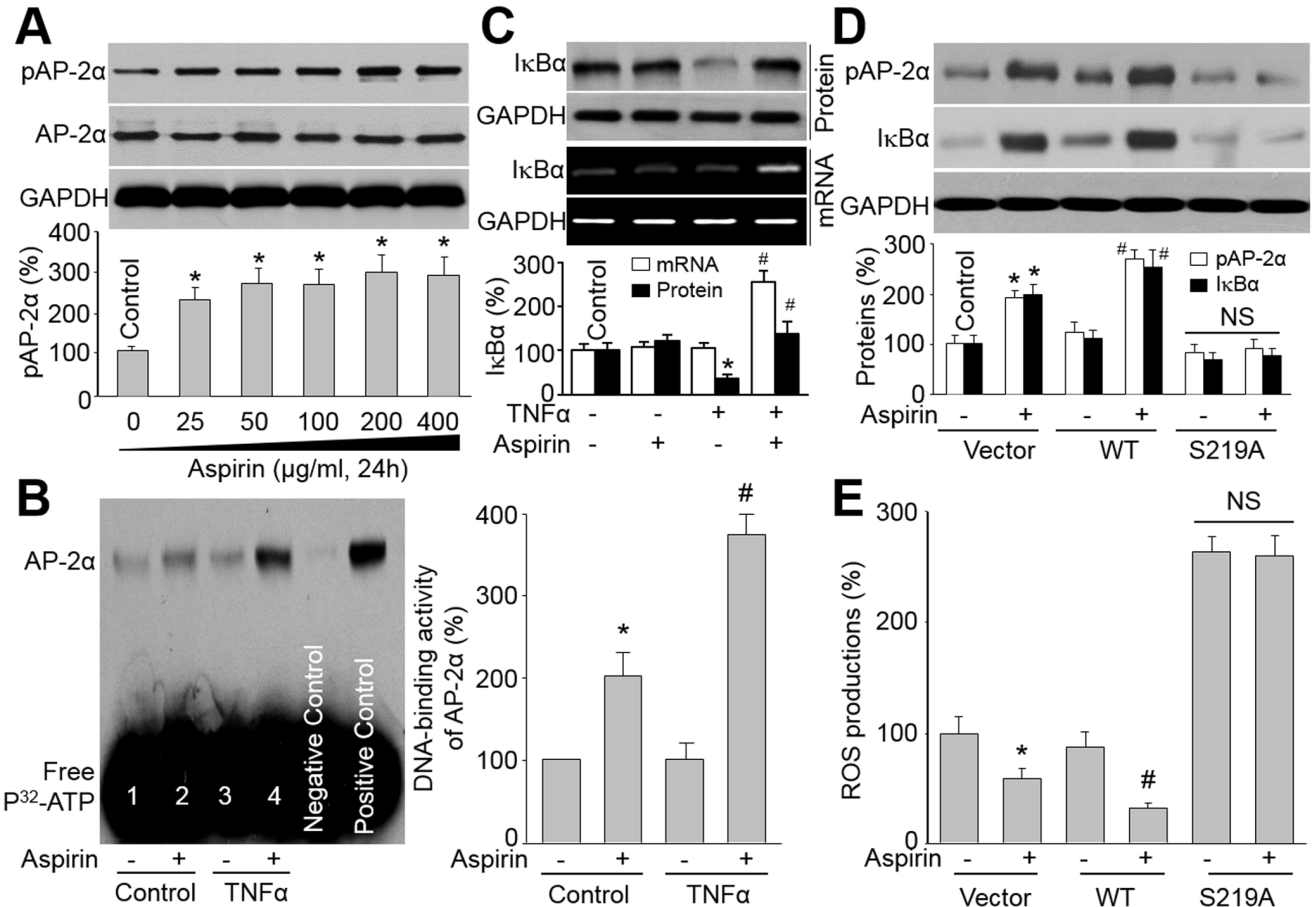
Aspirin *via* AP-2α upregulates IkBa levels in VSMCs **A.** Cultured VSMCs were treated with 25-400 μg/ml aspirin (24 hours). Cell lysates were immunoblotted with anti-phosphorylated AP-2α antibody. *P < 0.05 *vs*. Control. (B and C) VSMCs were treated with TNFα (10 ng/ml, 24 hours) plus aspirin (200 μg/ml). Cells were subjected to detect AP-2α DNA-binding activity by EMSA in B and the levels of IkBa protein and mRNA by Western blot or RT-PCR in C. *P < 0.05 *vs*. control. ^#^*P* < 0.05 *vs*. TNFα alone. (D and E) VSMCs were infected with adenovirus vector containing cDNA of WT-AP-2α or S219A-AP-2α for 48 hours. After that, cells were incubated with or without aspirin (200 μg/ml) for 24 hours. Cell lysates were harvested to assay protein level of IkBa by Western blot in D and ROS productions by DHE/HPLC in E. N is 3 in each group. *P < 0.05 *vs*. Vector alone. ^#^*P* < 0.05 *vs*. WT-AP-2α alone. NS indicates no significant difference.

We next addressed the molecular mechanism whereby aspirin can reduce the atherosclerotic burden and promote plaque stability. We cultured human VSMCs with increasing doses of aspirin and quantified phosphorylated AP-2α. As shown in Figure 5A, the serine 219 phosphorylation of AP-2α was increased by aspirin in a dose-dependent manner. To mimic the inflammatory microenvironment of vascular cells in atherosclerosis plaque, VSMCs were stimulated with tumor necrosis factor alpha (TNFα) to induce oxidative stress. AP-2α DNA-binding activity was assayed by EMSA. As indicated in Figure 5B, aspirin increased AP-2α DNA-binding activity in nuclear extracts from both resting and TNFα-stimulated endothelial cells, suggesting that aspirin functions as a potent AP-2α activator in VSMCs.

### Aspirin *via* AP-2α activation increases IkBα gene expression in VSMCs

The NF-kB-mediated NAD(P)H oxidase upregulation and oxidative stress, which is determined by IkBα [25], plays a critical role in atherosclerosis. Therefore, we hypothesized that aspirin *via* AP-2α activation increases IkBα gene expression to suppress NF-kB-mediated NAD(P)H oxidase-derived ROS productions in VSMCs. We then examined the levels of IkBα, a regulator of inflammation. The association between AP-2α activation and IκBα gene upregulation was tested by CHIP assay (Supplementary Figure S3A), indicating that there is an AP-2α binding site in the promoter/enhancer region of IκBα gene. In Figure 5C, TNFα dramatically decreased the IkBα protein level, while co-incubation of aspirin reversed TNFα-induced reduction of IkBα protein levels. Importantly, aspirin also increased IkBα mRNA in TNFα-treated cells, indicating that the upregulation of IkBα protein by aspirin may be through gene transcription.

To determine if activated AP-2α is required for aspirin-induced upregulation of IkBα, we infected endothelial cells with adenoviruses containing wild-type (WT) or mutant of AP-2α by replacing serine 219 to alanine (S219A). As shown in Figure 5D, aspirin increased the level of pS219-AP-2α in cells infected with plain vector or WT-AP-2α, but not in cells with overexpression of S219A-AP-2α mutant. Compared to plain vector, WT-AP-2α further enhanced aspirin-stimulated IkBα protein expression in cells. However, S219A-AP-2α abolished the effects of aspirin on IkBα protein expression in cells. Reversely, ROS production was not suppressed by aspirin in cells expressing S219A AP-2α (Figure 5E), indicating that activated AP-2α is required for aspirin's inhibitory effect on oxidative stress. In sum, activation of AP-2α, by serine 219 phosphorylation, is critical for suppression of oxidative stress by aspirin in VSMCs (Supplementary Figure S3B).

### Aspirin via AP-2α enhances IkBα protein and reduces oxidative stress in *Apoe^-/-^* mice

**Figure 6:**
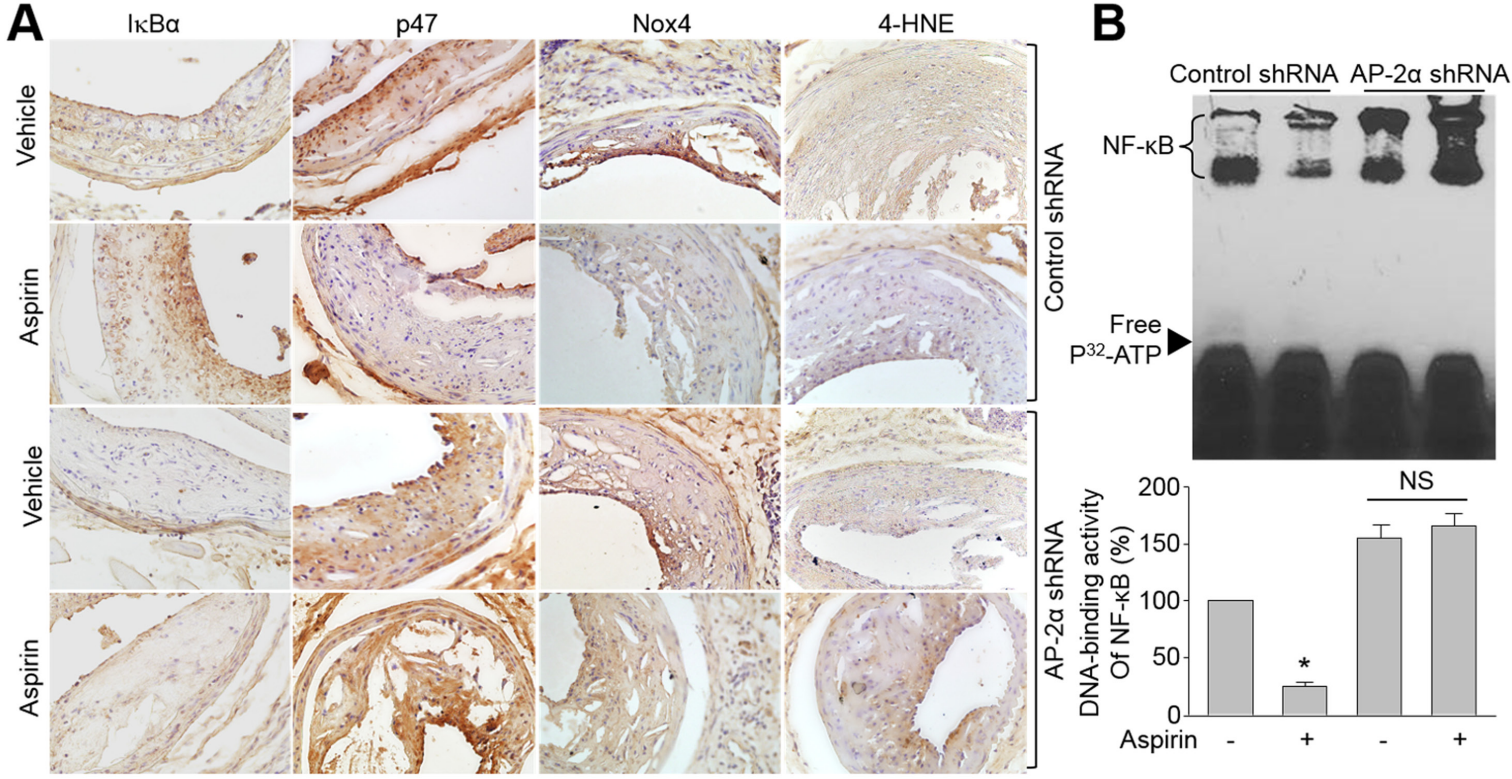
Knockdown of AP-2α abolishes the effects of aspirin on IkBα gene expression, NAD(P)H oxidase protein expressions, and oxidative stress in *Apoe^-/-^* mice with vulnerable atherosclerotic plaque The protocol and experimental designs were described in Supplementary Methods and Figure S2C. **A.** Protein expressions of IkBα, Nox4, p47, and 4-HNE by IHC staining were determined. The picture shown is a representative picture from 10-15 mice in each group. **B.** Quantitative analysis of NF-kB DNA-binding activity in mice carotid arteries assayed by EMSA. Data were expressed by mean ± SEM. N is 10-15 in each group. *P < 0.05 *vs*. control shRNA alone. NS indicates no significance.

The role of AP-2α on IkBα/NF-kB/NAD(P)H oxidase in mice was also investigated. In Figure 6A and 6B, aspirin dramatically increased IkBα protein level, inhibited NF-kB DNA-binding activity, and reduced the levels of p47, Nox4 and 4-HNE in carotid arteries from *Apoe^-/-^*- mice infected with control shRNA. The effects of aspirin on suppression of oxidative stress were further confirmed by detecting the levels of F2-isoprostanes, which was a non-invasive marker of oxidative stress [26], in urine and blood (Supplementary Figure S4A and S4B). Knockdown of AP-2α by shRNA bypassed these alterations induced by aspirin.

### Aspirin increases AP-2α phosphorylation and IkBα protein expression in humans

**Figure 7:**
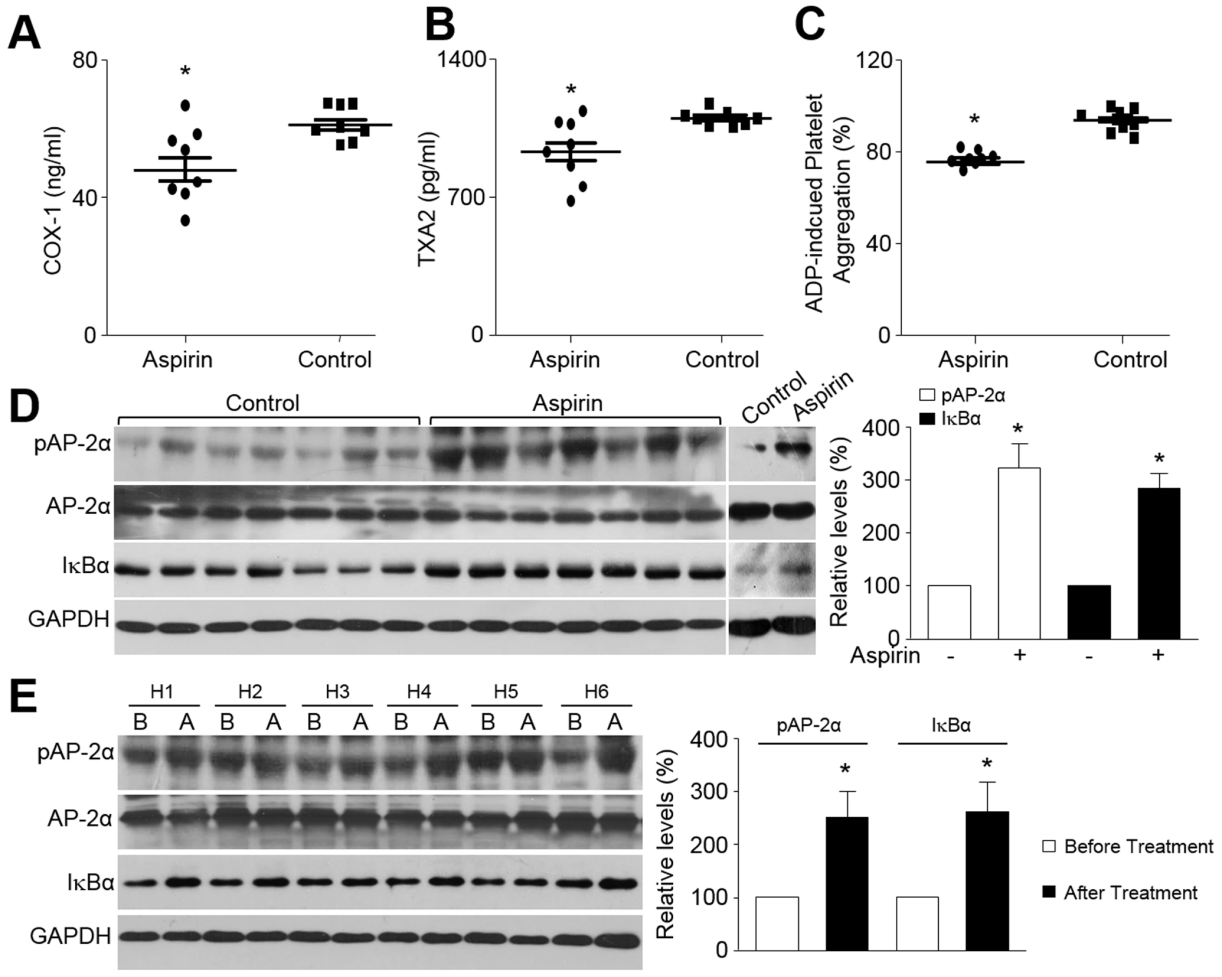
Aspirin activates AP-2α and increases IkBα protein expression in humans **A.**-**D.** The demographic data were shown in Supplementary Table S2. Blood were collected from human patients taking aspirin or not. Leucocytes were isolated from fresh blood and subjected to detect the COX-1 activity in A, the plasma TXA2 level in B, ADP-induced platelet aggregation in C, and the levels of phosphorylated AP-2α protein and total IkBα protein in D. Eight human subjects are in each group. *P < 0.05 *vs*. Control. **E.** The demographic data were shown in Supplementary Table S3. Six human subjects were recruited to taking aspirin (100 mg/day) for 4 weeks. Blood were collected before and after aspirin administration to detect the levels of phosphorylated AP-2α protein and total IkBα protein by Western blot to detect. H, Human subject. B, Before aspirin treatment. A, After aspirin treatment. *P < 0.05 *vs*. Before aspirin treatment. An unpaired or paired Student's *t*-test was applied for statistical comparisons between two groups.

Finally, to address if the conventional dose of aspirin activates AP-2α and upregulates IkBα protein level in humans, we assayed AP-2α phosphorylation and IkBα protein in humans. The compliance to aspirin treatment was assessed by determining the platelet cyclooxygenase 1 activity. As indicated in Figure 7A-7C, aspirin at the dose of 100 mg/day remarkably reduced both the COX-1 activity and the plasma TXA2 level in human subjects, and inhibited ADP-induced platelet aggregation. As expected, the levels of pAP-2α and IkBα protein in leucocytes were much higher in patients taking aspirin than those non-aspirin controls (Figure 7D). Due to probable interindividual differences, we also tested the levels of pAP-2α and IkBα protein in the same person. Similarly, the levels of pAP-2α and IkBα protein were increased after aspirin administration, compared to those before aspirin treatment (Figure 7E). Collectively, these data demonstrate that aspirin at conventional dose is able to activate AP-2α/IkBα pathway in humans, revealing the high clinical relevance of our experimental observations.

## DISCUSSION

In the present study, we have first provided the evidences to determine that AP-2α is a novel target of aspirin, and to show the important role of AP-2α in the development and instability of atherosclerotic plaque in all stages. In mice, aspirin suppressed the growth of atherosclerosis lesion and promoted the plaque stability. Importantly, all these effects of aspirin were abolished by knockdown of AP-2α *via* genetic approaches. Thus, we conclude that AP-2α is required for aspirin to suppress atherosclerosis.

An important finding of this study is that aspirin *via* AP-2α activation inhibits the growth and instability atherosclerotic plaques. We not only provided experimental evidences to indicate the action of aspirin to maintain the stability of advanced atherosclerotic plaque, but also identified a unique mechanism of aspirin on reducing acute coronary syndrome distinct from regular nonsteroidal anti-inflammatory drugs. It is well known that aspirin is one of the major preventive treatments against cardiovascular events to prevent vascular complications of atherosclerosis [27]. The traditional mechanism of action of aspirin is considered as inhibition of cyclooxygenases [20]. In this study, we observed that aspirin increased AP-2α serine 219 phosphorylation in cells and in mice. Importantly, the beneficial effects of aspirin on the growth and the stability of atherosclerosis plaque were abolished by knockdown of AP-2α *via* genetic approaches. Although it has been reported that AP-2α modulates the expression of ATP-binding cassette transporter A1 gene, which is a rate-limiting factor for high-density lipoprotein biogenesis [28, 29]. Moreover, AP-2α activation inhibits oxidative stress in human epithelial cells [30]. Our studies firstly indicate that AP-2α is critical in the progression of atherosclerosis and activated by aspirin.

In this study, we uncovered that AP-2α regulates IkBa gene expression. *In vitro* and *in vivo* knockdown of AP-2α by shRNA and expression of mutant S219A AP-2α reduced IkBa protein level. Under resting conditions, IkBα protein binds to NF-kB in cytoplasm. Upon stimulation by cytokines, IkBα undergoes rapid phosphorylation, ubiquitinylation, and subsequent degradation by the 26S proteasome, leading to NF-kB transactivation and consequent NAD(P)H oxidase expression [31]. Newly synthesized IkBα is an important source to supplier to balance the degradation of IkBα protein by cytokines [32]. We and others have previously observed that cytokines-induced NF-kB activation through 26S proteasome dependent IkBα degradation triggers oxidative stress and endothelial dysfunction [15, 33], which is a hallmark of early atherosclerosis. In fact, studies have reported that cardiovascular risk factors, such as hyperglycemia, enhanced 26S proteasome functionality in the early stage, not in the late stage [34]. Combining these evidences, we expect that 26S proteasome-dependent IkBα degradation might play an important role in the initiation of atherosclerotic plaque.

A limitation of this study is that the dose of aspirin at 20 or 50 mg/kg/day in animal studies appeared to be high compared with the dosages prescribed for humans. Because of the species differences in pharmacokinetics and pharmacodynamics of drug between human and mammal animals, the dose of aspirin in mice is not applied to human usage base on a body scale-adjusted scale. According to Chodera's reports [35], we general used 10 as the coefficient of biotransformation for drugs between human and mouse. Thus, 50 mg/kg of aspirin in mice would correspond to about 0.35 g aspirin for a 70 kg human subject. This is an acceptable dose in human because the doses of aspirin in humans are 100 mg/day for anti-thrombosis and 300-600 mg/day for anti-pain or anti-fever, which were recommend by ESC Guidelines. Importantly, we have confirmed that aspirin at a conventional dose of 100 mg/day increased AP-2α phosphorylation in human peripheral blood leukocytes, revealing the clinical relevance of this study.

In summary, we identified a novel molecular mechanism by which aspirin *via* AP-2α inhibits atherosclerosis in all stages. Thus, AP-2α might be a therapeutic target to prevention atherosclerosis-associated diseases, such as stroke or myocardial infarction caused by rupture of advanced atherosclerotic plaque.

## MATERIALS AND METHODS

A full description of materials and methods can be found in the online-only Supplementary Materials.

### Animal experiments and atherosclerotic lesion analysis

The collar placement was used to induce atherosclerotic vulnerable plaque in *Apoe^-/-^* mice. Briefly, a constrictive silastic tube was placed around the left common carotid artery near its bifurcation in mice fed with a high fat diet. Lentivirus infection was performed 4 weeks prior to aspirin administration (5-50 mg/kg/day). For analyzing the lesion area, the tissue was embedded in freezing medium and sectioned. Four consecutive sections were collected from each mouse and stained with Oil Red O or HE. The plaque vulnerable index in carotid artery was calculated according to the ratio of area I (Oil Red^+^ + CD68^+^) to area II (α-SMA^+^ + Collagen^+^).

### Patients and sample processing

Patients, with comparable baseline characteristics including blood cell and platelet counts, were enrolled to take aspirin (100 mg/kg/day) for 4 weeks or 2-6 months with informed consent. All patients were observed to ingest aspirin daily by phone call. The patients who finished the whole treatment without aspirin resistance were calculated in clinical investigations. The platelet cyclooxygenase 1 activity, plasma thromboxane A2 level, and ADP-induced platelet aggregation were measured to determine the effects of aspirin treatment. Leucocytes were isolated from blood and subjected to perform western blot analysis of pAP-2α and IkBα. These protocols complied with the Management Rules of the Chinese Ministry of Health and were approved by the Ethical Committee of Shandong University Qilu Hospital.

### Statistical analysis

Data were reported as mean ± SD or SEM. One-way ANOVA followed by Tukey *post-hoc* tests or Bonferroni correction was used for multiple comparisons. For statistical comparison between two groups, we used an unpaired or paired Student's t-test. Two-sided *P*-value < 0.05 was considered as significant.

## SUPPLEMENTARY MATERIALS FIGURES AND TABLES


