# Supplementary material for: Activation of activator protein 2 alpha by aspirin alleviates atherosclerotic plaque growth and instability in vivo
Source: Oncotarget. 2016 Jul 4;7(33):52729–39. doi: 10.18632/oncotarget.10400 (PMC5288144; doi:10.18632/oncotarget.10400)
Supplement: Supplementary file 1 [file oncotarget-07-52729-s001.pdf]

Activation of activator protein 2 alpha by aspirin alleviates atherosclerotic plaque growth and instability *in vivo*

Supplementary information

Methods

Reagents

Aspirin was purchased from Sigma Company (Cat: V900185). Polyclonal or monoclonal antibodies against p47, Nox4, GAPDH, α-SMA, p65, IκBα, AP-2α, 3-NT, and 4-HNE were obtained from Santa Cruz Biotechnology, Abcam, or Cell Signaling Company. All secondary antibodies were obtained Cell Signaling Technology. Antibody of pAP-2α Serine 219 was generated by Genscript Company. Dihydroethidium (DHE) was purchased from Molecular Probes. All PCR primers were purchased from Invitrogen. Other chemicals, if not indicated, were from Sigma-Aldrich (St. Louis, MO). All drug concentrations are expressed as the final molar concentration in the working buffer.

Patients and sample processing

For detecting the effects of aspirin in humans, we recruited 16 patients with coronary atherosclerotic heart disease taking aspirin or not. The profiles of these patients were shown in Supplementary Table S2 and S3. The blood was collected from these patients. Leucocytes were isolated from blood and subjected to biochemical analysis. These protocols complied with the Management Rules of the Chinese Ministry of Health and were approved by the Ethical Committee of Shandong University Qilu Hospital.

Animals and protocols of *in vivo* experiments

Apoe$^{-/-}$ mice were purchased from Jackson Laboratories (Bar Harbor, ME). Mice were housed in temperature-controlled cages with a 12-hrs light-dark cycle. The animal protocol was reviewed and approved by the University of Shandong Animal Care and Use Committee.

Our animal model consisted of three parts including high-fat diet induced aortic atherosclerosis, carotid collar induction of vulnerable plaques, and lentiviral knock down of AP-2α. The model of vulnerable plaque formation is shown in Supplementary Figure S1A. *Apoe*$^{-/-}$ mouse had a collar placed

around the left common carotid artery. In brief, a constrictive silastic tube (0.30-mm inner diameter, 0.50-mm outer diameter, and 2.5-mm long; Shandong Key Laboratory of Medical Polymer Materials, Jinan, China) was placed around the left common carotid artery near its bifurcation in male *Apoe*$^{-/-}$ mice at age of 8-12 weeks. Mice were then fed with a high fat diet (0.25% cholesterol and 15% cocoa butter) plus or minus aspirin administration (5, 20, 50 mg/kg/day) in drinking water for 8 weeks. At the end of experiment, all mice were sacrificed under anesthesia. The thoracic and abdominal aortas were collected for histological analysis of atherosclerotic lesion growth. The left common carotid arteries were collected for pathological analysis of atherosclerotic plaque stability.

In the second part of the animal study, as shown in Supplementary Figure S2A, male *Apoe*$^{-/-}$ mice at age of 8-12 weeks were infected with lentivirus containing negative control shRNA or AP-2α shRNA (once 4 weeks) via tail vein as described previously with light modifications[1] and received a high fat diet (0.25% cholesterol and 15% cocoa butter). 4 weeks later, aspirin (50 mg/kg/day per mouse) was added into drinking water and maintained for 8 weeks. At the end of experiment, mice were euthanized with an intraperitoneal injection of 0.8% pentobarbital sodium (60 mg/kg), followed by cervical dislocation. The aortas were collected for histological and molecular biological analysis.

In the third part of *in vivo* study, as shown in Supplementary Figure S2C, male *Apoe*$^{-/-}$ mice at age of 8-12 weeks had left carotid collar placement plus local lentivirus infection as described previously[2]. After surgery, mice were fed a high fat diet (0.25% cholesterol and 15% cocoa butter). 4 weeks later, mice received aspirin administration in drinking water (50 mg/kg/day) and injection of lentivirus (once every 4 weeks) and kept for 8 weeks. The left common carotid arteries were collected for pathological and molecular biological analysis.

Generation of shRNA construct and lentivirus production

Based on the protocol from Signaling Gateway, the shRNA cassette containing target sequence of AP-2α (GGAGAGCGAAGTCTAAGAATG) was designed. The cassette was subcloned into pEN-hH1c vector as described previously[3]. The pEN-hH1c vector containing the AP-2α shRNA cassette was combined

with an attR-containing vector pDSL-hpUP in an LR recombination reaction. The recombinant constructs pDSL-hpUP-AP-2α-shRNA were confirmed by DNA sequence analysis. The sequence of negative control shRNA is TTCTCCGAACGTGTCACGT. The lentivirus was produced by transiently transfecting HEK293T cells using SuperFect transfection reagent (Qiagen, USA) with three packing plasmid system (pGag/Pol, pRev, and pVSV-G).The virus-containing supernatant was collected 72 hours after transfection, and filtered through 0.45 mm filters (Millipore, USA), and stored at -80 °C. The titer of the viral vectors was determined by TCID50 (Tissue culture infective dose) method.

Cell cultures

Human vascular smooth muscle cells (VSMCs) from ATCC were grown in basal medium (Clonetics Inc. Walkersville, MD) supplemented with 2% FBS, penicillin (100 U/ml), and streptomycin (100 μg/ml). In all experiments, cells were between passages 3 and 8. All cells were incubated at 37°C in a humidified atmosphere of 5% CO_2 and 95% air. Cells were grown to 70-80% confluency before being treated with different agents.

Generation and infection of adenoviral infection to cells

To generate adenoviral vector containing Ad-S219A-AP-2α cDNA, we subcloned a human cDNA encoding full-length of AP-2α, which contained a substituted amino acid of serine 219 to alanine (S219A), into a shuttle vector (pShuttle CMV [cytomegalovirus]) as described previously[4]. VSMCs were infected with adenovirus overnight in medium supplemented with 2% FBS. The cells were then washed and incubated in fresh medium for an additional 24 hours before experimentation. These conditions typically produced an infection efficiency of at least 80%.

Western blot analysis

Cell lysates or tissue homogenates were subjected to western blot analysis, as described previously[5]. The protein content was assayed by BCA protein assay reagent (Pierce, USA). Protein of 20 μg was loaded to SDS-PAGE and then transferred to membrane. Membrane was incubated with a 1:1000 dilution of primary antibody, followed by a 1:5000 dilution of horseradish peroxidase–conjugated secondary antibody. Protein bands were visualized by ECL (GE Healthcare). The intensity (area × density) of the individual bands on

Western blots was measured by densitometry (model GS-700, Imaging Densitometer; Bio-Rad). The background was subtracted from the calculated area. We used control as 100%.

Detection of ROS

ROS productions in cultured cells were assayed by measuring the DHE fluorescence by HPLC with minor modification[6]. Briefly, cells were incubated with DHE (10 µM) for 30 min, washed, harvested and homogenized, and subjected to methanol extraction. HPLC was performed by using a C-18 column (mobile phase: gradient of acetonitrile and 0.1% trifluoroacetic acid) to separate and quantify oxyethidium (product of DHE and O_2^-) and ethidium (a product of DHE auto-oxidation). O_2^- production was determined by conversion of DHE into oxyethidine.

Electrophoretic mobility shift assay (EMSA) for AP-2α and NF-κB DNA-binding activities

Subcellular fractions were prepared by using NE-PER Nuclear and Cytoplasmic Extract kit (Cat78833) from PIERCE. EMSA were performed as described in a previous study[7]. AP-2α kit (AY1002) is from Panomics Company. NF-κB kit (E3050) is from Promeag Company.

ChIP assay for transcriptional factor and gene promoter binding

ChIP assays were performed by using a ChIP-IT kit (Upstate, 17-295), according to the manufacturer's protocol. 1×10^6 cells were seeded on a 10 cm dish. Proteins were cross-linked to DNA by adding formaldehyde directly to culture medium at a final concentration of 1% and incubating for 10 min at 37ºC. The cells were harvested in SDS lysis buffer and added protease inhibitors. Cell lysates were sonicated to shear DNA to lengths between 200 and 1000 bp. Sheared chromatin was precleared with protein G beads prior to incubation overnight at 4ºC with 4 µg of anti-AP-2α antibody, or control IgG antibody. Purified, immunoprecipitated chromatin fragments from IP samples were subjected to PCR. For IκBα promoter analysis, the primer sequences were as follows: Forward: 5' CTTTTTCTGGTCTGACTGGCT3' and reverse: 5' TGGAACATGGCGCGGACGAGCT 3'. PCR products were subjected to agarose gel electrophoresis and stained with ethidium bromide.

Reverse transcription PCR

Total cellular RNA was isolated using a Qiagen reagent and reverse transcribed to cDNA with specific antisense primers using the ThermoScript RT-PCR System protocol (Invitrogen). Samples (2 µl) of reverse transcribed product were PCR-amplified in a total volume of 25 µl with 10 pmoles each of forward and reverse primer. For IκBα mRNA analysis, the primer sequences were as follows: Forward: 5'GCTGTGATCCTGAGCTCCGAGAC3' and reverse: 5'CATCATAGGG CAGCTCGTCCTC3'. For GAPDH mRNA analysis, the primer sequences were as follows: Forward: 5'GCTGTGATCCTGAGCTCCGAGAC3' and reverse: 5'CATGTGGGCCAGGTCCACCAC3'.

Immunohistochemistry (IHC)

The thoracic aorta was fixed in 4% paraformaldehyde overnight, and then processed, embedded in paraffin, and sectioned at 4 µm. The deparaffinized, rehydrated section from thoracic aorta and cryosections from aortic root (5 µm) were microwaved in citrate buffer for antigen retrieval. Sections were incubated in endogenous peroxidase (DAKO) and protein block buffer, and then with primary antibodies indicated overnight at 4°C. Slides were rinsed with washing buffer and incubated with labeled polymer-horseradish peroxidase-antimouse/antirabbit antibodies followed by DAB+ chromogen detection (DAKO). After final washes, sections were counterstained with hematoxylin. All positive staining was confirmed by ensuring that no staining occurred under the same conditions with the use of non-immune rabbit or mouse control IgG.

Atherosclerotic lesion analysis

After being fed the Western diet for 8 weeks, the mice were fasted for 14 h and then were anesthetized and euthanized. The heart and aortic tissue were removed from the ascending aorta to the ileal bifurcation and placed in 4% paraformaldehyde for 16 h. After fixation, the adventitia was thoroughly cleaned under a dissecting microscope. For analyzing the lesion area in the aortic root, the heart was dissected from the aorta, embedded in Polyfreeze tissue freezing medium (Polysciences, Inc) and sectioned (5 µm thickness). Four consecutive sections were collected from each mouse and stained with

Oil Red O for neutral lipids, and counterstained with hematoxylin to visualize the nuclei. Plaques were captured under the Olympus microscope connected to a QImaging Retiga CCD camera. The aortic lesion size of each animal was obtained by the averaging of lesion areas in four sections from the same mouse. For analyzing the lesion area in the aortic arch, the intimal surface was exposed by a longitudinal cut from the ascending arch to 5 mm distal of the left subclavian artery to allow the lumen of the aortic arch to be laid flat. The aorta was rinsed for 5 min in 75% ethanol, stained with 0.5% Sudan IV in 35% ethanol and 50% acetone for 15 min, destained in 75% ethanol for 5 min, then rinsed with distilled water. Digital images of the aorta were captured under a stereomicroscopy, and the lesion area was quantified from the aortic arch to 5 mm distal of the left subclavian artery using Alpha Ease FC software (version 4.0 Alpha Innotech). The plaque vulnerable index was calculated according to the ratio of area I (Oil Red$^+$ + CD68$^+$) to area II (α-SMA$^+$ + Collagen$^+$) as described previously[8].

Measurement F_2-isoprostanes in urine and blood

The concentration of iPF$_2$α-VI in urine or blood was determined by LC-MS/MS as previously described[9]. In brief, 0.1 ml of 10 ng/ml deuterated internal standard (8iPF2α-d4; Cayman chemical, Ann Arbor, MI, USA) was added to 1 ml urine or blood. The sample was then subjected to solid phase extraction (Oasis HLB, Waters, Milford, MA, USA). The eluate was taken to dryness under a stream of nitrogen at room temperature, and afterwards redissolved in 100 μl 10% acetonitrile of which, 40 μl was injected on a reverse-phase XTerra MS C18 column (Waters, Milford, MA, USA; 3.5 μm, 2.1X100 mm). Urinary F2-isoprostanes were quantified using a Quattro Micro (Waters) mass spectrometer. To calculate the iPF$_2$α-VI concentration, the analyte to internal standard peak area ratio was compared with a standard curve from 2 to 16 ng/ml iPF$_2$α-VI (Cayman chemical, Ann Arbor, MI, USA). The intra-run coefficient of variation (CV) was 4.8% and the inter-run CV was 10.1%.

Statistical analysis

All quantitative data are reported as mean ± SEM. and were analyzed using a two-way ANOVA. Bonferroni corrections were applied to multiple comparisons and $P<0.05$ was considered significant.

References

1. Jalkanen J, Leppanen P, Pajusola K, Narvanen O, Mahonen A, Vahakangas E, Greaves DR, Bueler H, Yla-Herttuala S. Adeno-associated virus-mediated gene transfer of a secreted decoy human macrophage scavenger receptor reduces atherosclerotic lesion formation in ldl receptor knockout mice. *Molecular therapy : the journal of the American Society of Gene Therapy*. 2003;8:903-910
2. Li JJ, Meng X, Si HP, Zhang C, Lv HX, Zhao YX, Yang JM, Dong M, Zhang K, Liu SX, Zhao XQ, Gao F, Liu XL, Cui TX, Zhang Y. Hepcidin destabilizes atherosclerotic plaque via overactivating macrophages after erythrophagocytosis. *Arterioscler Thromb Vasc Biol*. 2012;32:1158-1166
3. Godec J, Cowley GS, Barnitz RA, Root DE, Sharpe AH, Haining WN. Inducible rnai in vivo reveals that the transcription factor batf is required to initiate but not maintain cd8+ t-cell effector differentiation. *Proc Natl Acad Sci U S A*. 2015;112:512-517
4. Wang S, Zhang M, Liang B, Xu J, Xie Z, Liu C, Viollet B, Yan D, Zou MH. Ampkalpha2 deletion causes aberrant expression and activation of nad(p)h oxidase and consequent endothelial dysfunction in vivo: Role of 26s proteasomes. *Circ Res*. 2010;106:1117-1128
5. Wang S, Xu J, Song P, Viollet B, Zou MH. In vivo activation of amp-activated protein kinase attenuates diabetes-enhanced degradation of gtp cyclohydrolase i. *Diabetes*. 2009;58:1893-1901
6. Wang S, Xu J, Song P, Wu Y, Zhang J, Chul Choi H, Zou MH. Acute inhibition of guanosine triphosphate cyclohydrolase 1 uncouples endothelial nitric oxide synthase and elevates blood pressure. *Hypertension*. 2008;52:484-490
7. Aravindan N, Mohan S, Herman TS, Natarajan M. Nitric oxide-mediated inhibition of nfkappab regulates hyperthermia-induced apoptosis. *J Cell Biochem*. 2009;106:999-1009
8. Dong M, Yang X, Lim S, Cao Z, Honek J, Lu H, Zhang C, Seki T, Hosaka K, Wahlberg E, Yang J, Zhang L, Lanne T, Sun B, Li X, Liu Y, Zhang Y, Cao Y. Cold exposure promotes atherosclerotic plaque

growth and instability via ucp1-dependent lipolysis. *Cell Metab*. 2013;18:118-129

9. Fischer SG, Perez RS, Nouta J, Zuurmond WW, Scheffer PG. Oxidative stress in complex regional pain syndrome (crps): No systemically elevated levels of malondialdehyde, f2-isoprostanes and 8ohdg in a selected sample of patients. *Int J Mol Sci*. 2013;14:7784-7794

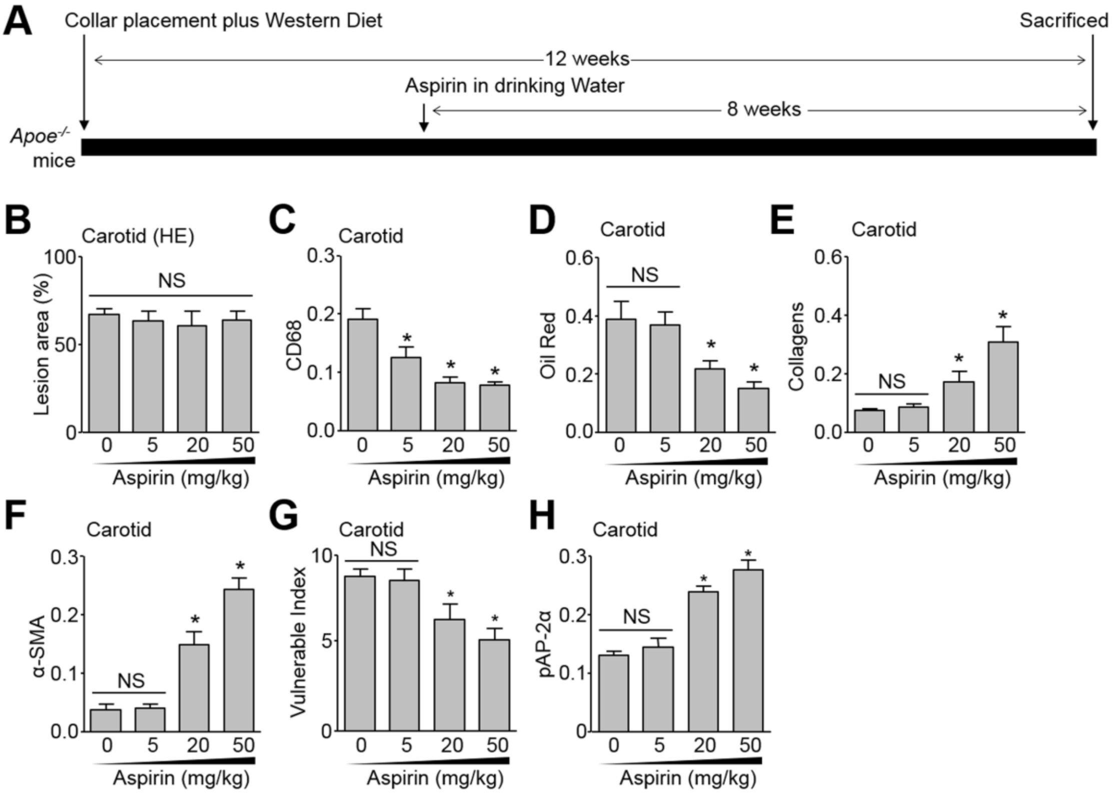

Supplementary Figure S1. Quantitative analysis of the aspirin-induced effect of atherosclerotic plaque growth and vulnerability in *Apoe*$^{-/-}$ mice. **(A)** The protocol of collar placement and aspirin administration in *Apoe*$^{-/-}$ mice. At the end of experiments, all mice were sacrificed under anaesthesia. (**B**) Quantitative data for sizes of atherosclerotic lesion in left common carotid arteries. (**C**) Quantitative data for macrophages numbers. (**D**) Quantitative data for lipid content. (**E**) Quantitative data for collagens. (**F**) Quantitative data for vascular smooth muscle cells numbers. (**G**) Vulnerability index was determined by using the ration of CD68^{+} (%) plus Oil Red (%) to α-SMA (%) plus collagen (%). (**H**) Quantitative data for phosphorylated AP-2α. N is 10-15 in each group. $^{*}P<0.05$ vs. Control (Aspirin at 0). NS indicates no significant difference.

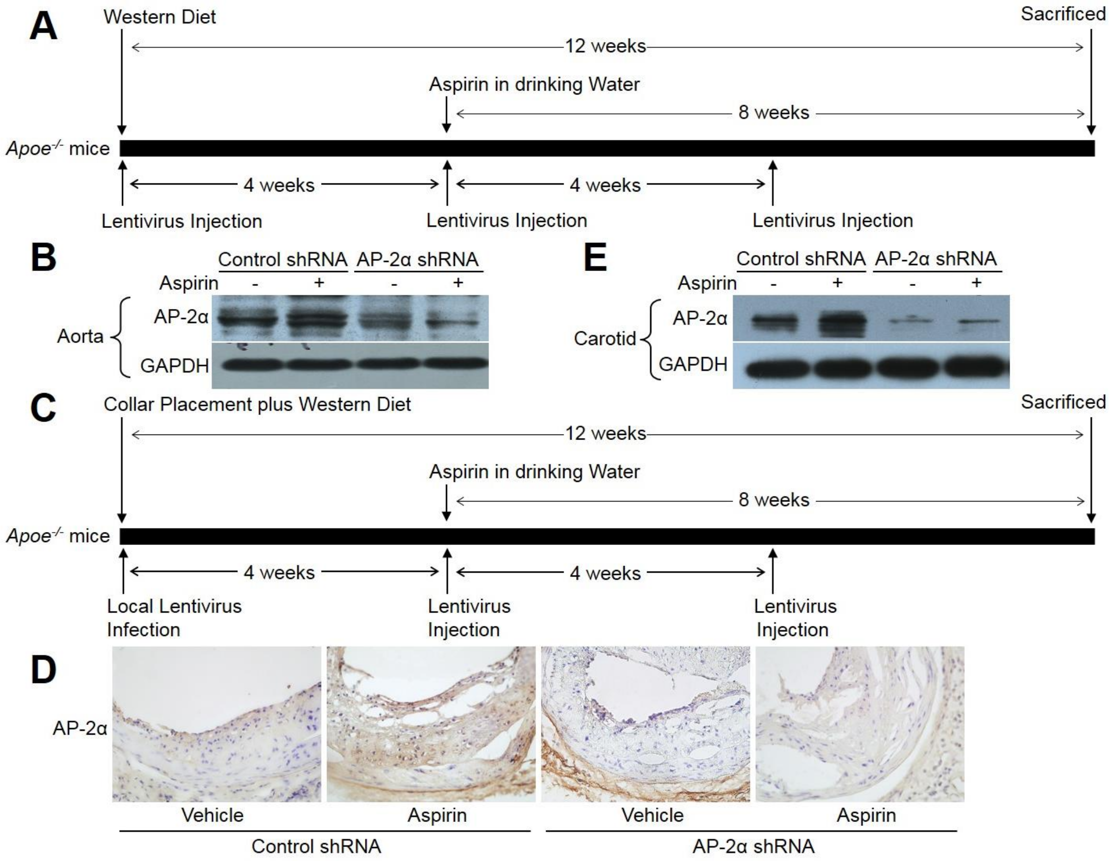

Supplementary Figure S2. Infection of lentivirus containing control or AP-2α shRNA silences AP-2α protein expression in aorta and carotid in *Apoe*$^{-/-}$ mice. (**A** and **B**) The protocol of lentivirus infection and aspirin administration in *Apoe*$^{-/-}$ mice fed with high fat diet was illustrated in **A**. 4 weeks later after injection, aortic roots were isolated from mice to detect AP-2α protein expression by western blot in **B**. (**C**-**E**) The protocol of collar placement, lentivirus infection, and aspirin administration in *Apoe*$^{-/-}$ mice was illustrated in **C**. 4 weeks later after infection, carotids were isolated from mice to detect AP-2α protein expression by IHC in **D** and by western blot in **E**.

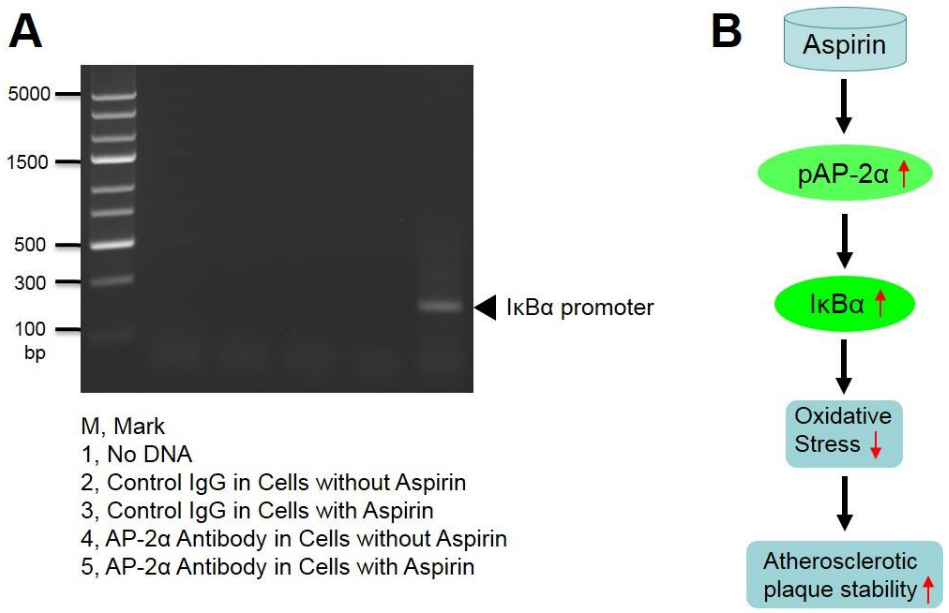

Supplementary Figure S3. Aspirin increases the binding of AP-2α to the promoter of IκBα in VSMCs. (**A**) VSMCs were incubated with aspirin (200 μg/ml) for 24 hours. Binding of AP-2α on the promoter of IκBα analyzed by ChIP assay. The picture is a representative of three individual experiments. (**B**) Proposed mechanisms of the aspirin-induced suppressive effects on the growth and instability of atherosclerotic plaque.

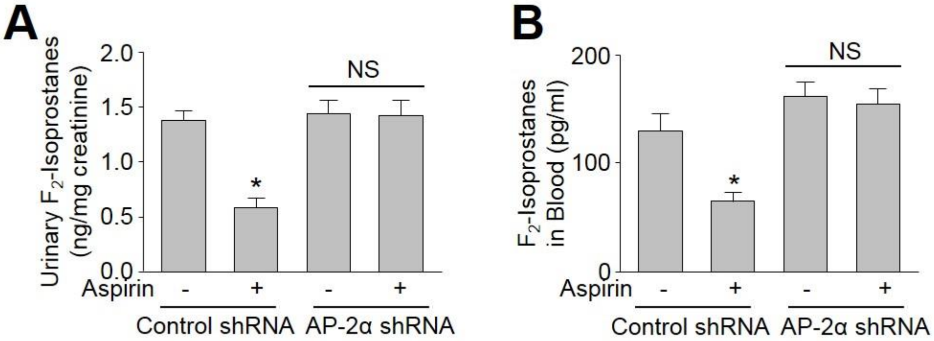

Supplementary Figure S4. Knockdown of AP-2α abolishes the effects of aspirin on oxidative stress in *Apoe*$^{-/-}$ mice with vulnerable atherosclerotic plaque. The protocol and experimental designs were described in Supplementary Methods and Figure S2C. (**A**) Urine levels of F_2-isoprostanes. (**B**) Blood levels of F_2-isoprostanes. Data were expressed by mean ± SEM. N is 10-15 in each group. $^{*}P<0.05$ vs. control shRNA alone. NS indicates no significance.

Supplementary Table S1. Serum lipids, blood glucose, and monocyte count in AP-2α-knockdown mice

Groups	TC(mM)	TG(mM)	GLU(mM)	Monocytes (10^9/L)
Control shRNA	10.73 ± 1.35	1.74 ± 0.22	6.80 ± 0.63	1.94 ± 0.76
Control shRNA + Aspirin	9.97 ± 0.61	1.67 ± 0.09	5.76 ± 1.28	1.28 ± 0.57
AP-2α shRNA	10.25 ± 1.55	1.96 ± 0.28	5.69 ± 0.61	1.83 ± 0.66
AP-2α shRNA + Aspirin	10.64 ± 1.49	1.88 ± 0.08	5.16 ± 0.87	1.75 ± 0.91

TC, total cholesterol; TG, triglycerides; GLU; glucose. N is 12 in each group. Data were expressed by mean ± s.e.m. **P*<0.05 vs. Control shRNA.

Supplementary Table S2. Demographic data for control individuals and patients taking aspirin

ID	Gender	Ages (years old)	Aspirin (dose and time)	WBC (10^9/L)	Platelet (10^9/L)
1	Male	44	None	5.93	118
2	Male	41	None	6.18	267
3	Male	48	None	5.36	186
4	Female	62	None	4.17	195
5	Male	57	None	7.08	214
6	Female	65	None	8.24	227
7	Male	56	None	6.92	176
8	Female	70	None	7.84	254
9	Male	45	100 mg/day for 3 months	6.57	193
10	Male	47	100 mg/day for 6 months	5.09	207
11	Female	64	100 mg/day for 4 months	9.13	238
12	Male	67	100 mg/day for 2 months	8.26	190
13	Female	58	100 mg/day for 2 months	6.17	217
14	Male	72	100 mg/day for 3 months	7.36	137
15	Male	49	100 mg/day for 3 months	8.29	202
16	Female	77	100 mg/day for 3 months	5.37	183

Supplementary Table S3. Demographic characteristics of the study participants

ID	Gender	Ages (years old)	Body Weight (kg)	Aspirin (dose and time)
1	Male	40	75	100 mg/day for 4 weeks
2	Male	35	70	100 mg/day for 4 weeks
3	Male	25	60	100 mg/day for 4 weeks
4	Female	25	55	100 mg/day for 4 weeks
5	Female	30	60	100 mg/day for 4 weeks
6	Female	35	65	100 mg/day for 4 weeks
